# Positron emission tomography imaging with ^89^Zr-labeled anti-CD8 cys-diabody reveals CD8^+^ cell infiltration during oncolytic virus therapy in a glioma murine model

**DOI:** 10.1038/s41598-021-94887-x

**Published:** 2021-07-28

**Authors:** Benjamin B. Kasten, Hailey A. Houson, Jennifer M. Coleman, Jianmei W. Leavenworth, James M. Markert, Anna M. Wu, Felix Salazar, Richard Tavaré, Adriana V. F. Massicano, G. Yancey Gillespie, Suzanne E. Lapi, Jason M. Warram, Anna G. Sorace

**Affiliations:** 1 Department of Neurosurgery, University of Alabama at Birmingham, Birmingham, AL USA; 2 Department of Radiology, University of Alabama at Birmingham, Volker Hall G082, 1670 University Boulevard, Birmingham, AL 35294 USA; 3 O’Neal Comprehensive Cancer Center, University of Alabama at Birmingham, Birmingham, AL USA; 4 Department of Immunology and Theranostics, City of Hope, Duarte, CA USA; 5 Department of Molecular and Medical Pharmacology, Crump Institute for Molecular Imaging, David Geffen School of Medicine at University of California Los Angeles, Los Angeles, CA USA; 6 Regeneron Pharmaceuticals, Inc., Tarrytown, NY USA; 7 Department of Otolaryngology, University of Alabama at Birmingham, Volker Hall G082, 1670 University Boulevard, Birmingham, AL 35294 USA; 8 Department of Biomedical Engineering, University of Alabama at Birmingham, Birmingham, AL USA

**Keywords:** Cancer, Biomarkers, Molecular medicine, Oncology

## Abstract

Determination of treatment response to immunotherapy in glioblastoma multiforme (GBM) is a process which can take months. Detection of CD8^+^ T cell recruitment to the tumor with a noninvasive imaging modality such as positron emission tomography (PET) may allow for tumor characterization and early evaluation of therapeutic response to immunotherapy. In this study, we utilized ^89^Zr-labeled anti-CD8 cys-diabody-PET to provide proof-of-concept to detect CD8^+^ T cell immune response to oncolytic herpes simplex virus (oHSV) M002 immunotherapy in a syngeneic GBM model. Immunocompetent mice (n = 16) were implanted intracranially with GSC005 GBM tumors, and treated with intratumoral injection of oHSV M002 or saline control. An additional non-tumor bearing cohort (n = 4) receiving oHSV M002 treatment was also evaluated. Mice were injected with ^89^Zr-labeled anti-CD8 cys-diabody seven days post oHSV administration and imaged with a preclinical PET scanner. Standardized uptake value (SUV) was quantified. Ex vivo tissue analyses included autoradiography and immunohistochemistry. PET imaging showed significantly higher SUV in tumors which had been treated with M002 compared to those without M002 treatment (p = 0.0207) and the non-tumor bearing M002 treated group (p = 0.0021). Accumulation in target areas, especially the spleen, was significantly reduced by blocking with the non-labeled diabody (p < 0.001). Radioactive probe accumulation in brains was consistent with CD8^+^ cell trafficking patterns after oHSV treatment. This PET imaging strategy could aid in distinguishing responders from non-responders during immunotherapy of GBM.

## Introduction

Glioma is associated with a median overall survival of less than 18 months despite multi-modal treatment with conventional, clinically approved therapy (e.g., surgery, chemo-radiotherapy)^1^. Various immunotherapy strategies, including checkpoint inhibitors and oncolytic herpes simplex virus (oHSV) therapy, are being explored in attempts to improve the prognosis for glioma^2–9^. oHSV has been shown to directly kill tumor cells as the virus selectively replicates within and lyses malignantly transformed cells^10,11^. The expression of viral antigens and release of tumor antigens stimulates innate and adaptive immune cells to traffic to the tumor and potentially evoke an antitumor immune response^12–14^. Treatment of glioma patients with conditionally replicating, γ_1_*34.5-*deleted oHSV G207 was found to be safe and provide evidence of anti-tumor efficacy in previous clinical trials^15–17^. However, similar to other immunotherapies, a significant proportion of treated patients do not exhibit a clinical response to oHSV and show continued tumor progression after treatment intervention^15^. M002 is an alternative γ_1_*34.5-*deleted oHSV that also expresses murine protein interleukin-12 (IL-12), which stimulates immune cell activity; M002 has shown greater anti-tumor and immune-stimulatory effects than G207 in preclinical studies^11,18^. The human version of M002, M032, is currently being investigated in a phase I clinical trial in patients with malignant brain tumors^19^.

Immune cell infiltration into the tumors can resemble tumor progression (psuedoprogression) during conventional radiographic and molecular imaging with contrast-enhanced magnetic resonance imaging and 2-deoxy-2[^18^F]fluoro-d-glucose (FDG)—positron emission tomography (PET) imaging. Therefore, these standard-of-care anatomical and molecular imaging approaches have not been able to reliably evaluate or monitor tumor response at early time points during immunotherapy treatment^20,21^, requiring months of patient treatment and multiple imaging sessions to determine if the patient is responding to or progressing on therapy. Obtaining repeat biopsies of central nervous system (CNS) tissues to distinguish between immune cell infiltration and tumor cell proliferation is not always clinically feasible. Therefore, there remains an unmet clinical need to effectively characterize immune cell infiltration into tumors and distinguish between tumor response and tumor resistance in glioma patients treated with oHSV immunotherapy.

One approach to address the clinical need of monitoring and predicting response is to employ targeted imaging to detect the in vivo localization of an immune cell population that plays an essential role in the antitumor immunotherapy response. Cytotoxic CD8^+^ T cells represent an attractive immune cell population to target for imaging responses to immunotherapy. Activated CD8^+^ cytotoxic T cells are among the main mediators of the tumor-specific immunotherapy response during treatment using oHSV or alternative types of immunotherapy^22^. These immune cells recognize and destroy target tumor cells via release of cytokines, perforins, and granzymes. Preclinical and clinical studies have shown that the relative increase in CD8^+^ T cells that localize in a tumor during immunotherapy correlate with treatment response^23^. Immunohistochemistry and flow cytometry analyses following oHSV treatment have shown significant accumulation of CD8^+^ T cells in CNS tumors during preclinical studies^10,11,24,25^, and in blood and tumor tissue collected during trials in human patients^15,26^. Various CD8-targeted imaging agents have been used for in vivo PET in preclinical models of malignancy treated with immunotherapy^27–30^. Studies employing a radiolabeled anti-mouse CD8 cys-diabody (cDb) in several models of non-CNS malignancies demonstrated correlation between the in vivo PET signal and the number of CD8^+^ cells present in the tumors^28^. This agent was able to distinguish between tumors that eventually responded to checkpoint immunotherapy and tumors that were resistant to treatment^28,31^. However, CD8-targeting probes for PET imaging have not yet been evaluated in glioma models treated with oHSV or other types of immunotherapy.

In this contribution, we demonstrate proof-of-concept for CD8-targeted PET in vivo imaging in preclinical, orthotopic glioma models during treatment with oncolytic virus immunotherapy. Mice with syngeneic orthotopic glioma tumors (GSC005) were imaged with ^89^Zr-labeled anti-CD8 cys-diabody PET to identify uptake in tumor-bearing and normal brains to characterize immune cell infiltration in response to oHSV. Biological validation with immunohistochemistry and autoradiography was utilized to validate imaging findings.

## Materials and methods

### General

All general reagents were from commercial suppliers (Thermo Fisher, Waltham, MA, USA; Sigma, St. Louis, MO, USA) unless specified otherwise. The anti-mouse CD8α (Lyt2) 169 cDb was produced and conjugated to the maleimide-desferrioxamine (malDFO) chelate to generate malDFO-169 cDb for radiolabeling as previously described^28^.

### Cell culture

TK-1 murine lymphoma cells (CD8^+^) were obtained from the American Tissue Type Collection (Manassas, VA) and cultured in suspension in RPMI 1640 medium with 2 mM l-glutamine, 1.5 g/L sodium bicarbonate, 4.5 g/L glucose, 10 mM HEPES, 1.0 mM sodium pyruvate, 0.1 mM non-essential amino acids, 0.05 mM 2-mercaptoethanol, and 10%; fetal bovine serum (FBS). D-54MG cells gifted from Darell D. Bigner (Duke University, Durham NC) and U-87MG cells obtained from the American Tissue Type Collection (Manassas, VA) were cultured as adherent monolayers in Dulbecco’s Modified Eagle’s Medium supplemented with 10% fetal bovine serum and prophylactic plasmocin. Previously characterized murine glioma GSC005 cells^32,33^ gifted from Dr. Inder Verma of the Salk Institute were maintained in Corning tissue culture plasticware (Corning, Inc., Corning, NY) as neurospheres in Dulbecco’s Modified Eagle’s Medium/F12 containing N2 supplement, 2 mM l-glutamine, penicillin, streptomycin, 20 ng/mL recombinant human epidermal growth factor, 20 ng/mL recombinant human fibroblast growth factor, and 5 µg/mL heparin. Prior to tumor cell implantation in mice, the GSC005 neurospheres were harvested and dissociated into a single cell suspension with TrypLE™ Express (Gibco) and gentle trituration. Dissociated cells were centrifuged, washed and re-suspended in phosphate-buffered saline (PBS).

### ^89^Zr radiolabeling

[^89^Zr]-oxalate was produced at the University of Alabama at Birmingham Cyclotron Facility. The ^89^Zr-oxalate solution was diluted with an equal volume of 0.5 M pH 7 HEPES followed by 2 M NaOH solution to achieve a pH of ~ 7. The malDFO-169 cDb conjugate was added to give a specific activity of 180–220 MBq/mg in the reaction and mixed for 1 h at 37 °C using a thermomixer with shaking (600–800 rpm), and then purified using 7 K MWCO Zeba Spin Desalting Columns (Thermo Scientific) equilibrated in PBS. The radiolabeling efficiency of the crude reaction and radiochemical purity following purification were analyzed as follows: 1 µL aliquots of the appropriate solutions were spotted on Whatman #1 filter paper strips and allowed to dry. The strips were developed in 20 mM sodium citrate pH 6.5 containing 0.15 mM diethylenetriaminepentaacetic acid and allowed to dry. The relative activity at the solvent front (unconjugated ^89^Zr) and at the origin ([^89^Zr]-malDFO-169 cDb) of the strips was determined by imaging activity-mediated luminescence while exposing the strips to a β intensifying Kodak Biomax Transcreen. Luminescence was acquired using an IVIS Lumina III instrument (Perkin Elmer Inc., Waltham, MA). Background-corrected luminescence counts at the origin (O) and at the solvent front (SF) were used to calculate % labeling efficiency or % radiochemical purity by the equation: O/(O + SF) × 100%. A specific activity of 0.16–0.19 MBq/µg [^89^Zr]-malDFO-169 cDb was used in the experiments below.

### Immunoreactivity assay

The immunoreactivity of [^89^Zr]-malDFO-169 cDb was performed as previously described^28^ with minor modifications. Briefly, 2–3 replicate tubes containing 40 million TK-1 cells, or containing human glioma cells (U-87MG, D-54MG) as controls for non-specific binding, were incubated in suspension in 1 mL PBS containing 1% FBS and 3.5 µg/mL [^89^Zr]-malDFO-169 cDb for 1–2 h at room temperature on a rotator. The cells were pelleted by centrifugation (1500 × *g*, 4 min), washed twice with 1 mL PBS (suspending and re-centrifuging the cells during each wash), and the activity in the cell pellet was counted. Immunoreactivity was calculated by comparing the cell-bound activity in the pellet to the initially applied activity, measured in separate tubes in triplicate.

### Animal experimentation

**Figure 1:**
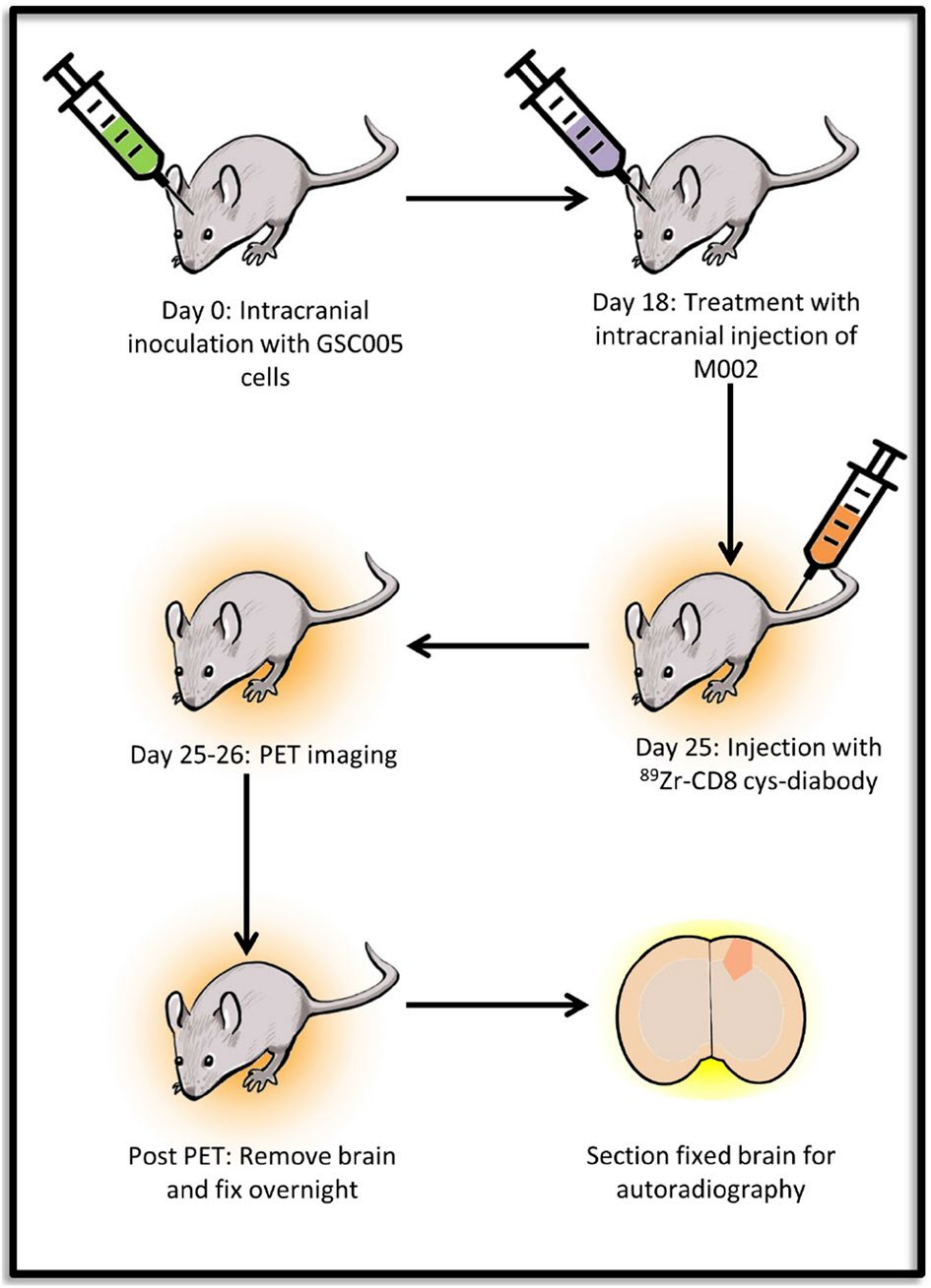
Experiment workflow. On day 0, C57BL6N mice underwent intracranial injection of 5 × 10^4^ of GSC005 glioma cells. On day 18–20 post inoculation, mice were treated with an intracranial injection of 1 × 10^7^ PFU of M002 oHSV or saline control. Seven days after M002, mice were injected with ~ 1.6 MBq of ^89^Zr labeled CD8 diabody. Mice were imaged at 1, 4 and 24 h post injection. After the 24 h imaging time point, brains were removed and processed for autoradiography. This figure was generated using Microsoft PowerPoint version 15.0.

Animal studies were approved by the Institutional Animal Care and Use Committee at the University of Alabama at Birmingham and performed in compliance with guidelines from the Public Health Service Policy and Animal Welfare Act of the United States. The studies are in accordance with ARRIVE guidelines. The experiment workflow for the mouse studies is shown in Fig. 1. C57BL6N mice (N = 20 total), obtained from Charles River Laboratories (Hartford, CT), between 6 and 8 weeks of age were used in the following studies. Orthotopic syngeneic GSC005 glioma tumors were generated using procedures similar to those previously described^11^. Briefly, female mice (N = 16) were implanted intracranially (i.c.) in the right cerebral hemisphere with 5 × 10^4^ GSC005 cells. These tumor-bearing mice were randomly divided into the following study groups: “GSC005 + M002” (n = 8 mice/group), “GSC005 + M002 + Block” (n = 4 mice/group), and “GSC005 + saline” (n = 4 mice/group). I.c. treatment with oHSV M002 in mice bearing orthotopic syngeneic GSC005 glioma tumors was performed to stimulate anti-tumor immunotherapy, which was expected to cause localization of CD8^+^ T cells to the tumor. oHSV M002 (1 × 10^7^ plaque-forming units, PFU) or vehicle control (saline) was injected intratumorally, through the i.c. burr hole, in mice in the respective groups (M002 in “GSC005 + M002” and “GSC005 + M002 + block” study groups; saline in “GSC005 + saline” study group) following tumor formation (18–20 days after tumor cell implantation). A separate control group of age-matched naïve mice (saline only) was injected i.c. in the right cerebral hemisphere with 1 × 10^7^ PFU M002; this control group (n = 4 mice) was referred to as “No tumor + M002”.

### PET/Computed Tomography (CT) in vivo imaging and biodistribution

A previous study indicated peak influx of CD8^+^ cells in orthotopic glioma tumors occurs at 6–8 days after oHSV treatment^11^. Thus, in the present study, mice were given an intravenous (i.v.) administration (tail vein) of [^89^Zr]-malDFO-169 cDb (~ 11 µg, ~ 1.6 MBq) 7 days after i.c. injection with oHSV M002 or saline (Fig. 1). Tumors ranged from 1.5 to 3 mm in diameter at this time in the experiment. Mice in the “GSC005 + M002 + Block” study group were co-injected with [^89^Zr]-malDFO-169 cDb and 65 µg unlabeled 169 cDb to block in vivo binding of the radiotracer to CD8. Approximately 1, 4, and 24 h after injection, mice were imaged by PET (energy window 350–650 keV; 10 min acquisition for 1 and 4 h time points, 20 min for 24 h time point) and CT (voltage 80 kVp, current 150 μA, 720 projections) acquired on a GNEXT PET/CT small animal scanner (Sofie Biosciences, Culver City, CA). Following the last imaging time point (24 h), anesthetized mice were euthanized by cervical dislocation, and blood was collected via cardiac puncture. Isoflurane was used for anesthesia. Whole brains, spleen, heart, liver, stomach, small intestine, large intestine (with contents), cecum, lungs, kidneys, leg muscle, and bone (femur) were resected, weighed, and counted on a gamma counter (1480 Wizard^2^, Perkin Elmer, Shelton, CT). Percent uptake of the injected dose per gram (%ID/g) was calculated by comparing the tissue activity to solutions with known activity of ^89^Zr. The mean brain-to-blood ratio for each group of mice was determined from the ex vivo biodistribution data using the formula: (brain %ID/g)/(blood %ID/g).

### PET/CT image analyses

The PET images were reconstructed using a 3D-Ordered Subset Expectation Maximization algorithm (24 subsets and 3 iterations), with random, attenuation, and decay correction*.* The CT images were reconstructed using a Modified Feldkamp Algorithm. Reconstructed images were analyzed using VivoQuant software (version 3.5patch 2, Invicro, LLC, Boston, MA). Standardized uptake value (SUV) was quantified by manually drawing 3D regions of interest (ROIs) using CT anatomical guidance over brain regions in the right hemisphere corresponding to tumor or oHSV injection, and in the adjacent (left) normal hemisphere. Activity was normalized to injected dose, mouse weight, and radioactive decay. Mean and standard deviation of the SUVs were determined using the formula: SUV = [(MBq/mL) × (animal wt. (g))/injected dose (MBq)]. SUV_mean_ was defined as the average SUV in a specific ROI. SUV_max_ was defined as the single voxel containing the maximum SUV value in the ROI. SUV_peak_ was defined as the average SUV of the 3 × 3 × 3 voxel grid containing the SUV_max_ voxel. The tumor SUV ratio (SUVr) was defined as the tumor SUV_mean_ divided by the contralateral brain hemisphere SUV_mean_.

### Processing of resected brains

Resected whole brains from tumor-bearing mice were fixed in 10% buffered formalin overnight and gross sectioned serially into 1 mm coronal slices. Gross tissue slices were placed over a β intensifying Kodak Biomax Transcreen and the activity-mediated luminescence in the slices was imaged for 10 min using an IVIS Lumina III instrument (Perkin Elmer, Shelton, CT). Integrated instrument software was used to determine mean ratio of luminescence in a region of interest (ROI) placed over the tumor divided by an ROI of the same size placed over the adjacent normal brain. Brain slices containing tumor and adjacent normal brain without tumor were then dehydrated in 70% ethanol and processed for embedding in paraffin and further sectioning (5 µm). These tissue sections were mounted on charged glass slides and stained using hematoxylin and eosin (H&E) or for CD8 immunohistochemistry.

### CD8 immunohistochemistry

Mounted tissues were deparaffinized in an EZ-DEWAX bath two times for 5 min. Antigen retrieval was achieved by heating in citrate buffer (Thermo Fisher) for 10 min at 100 °C. Slides were then cooled at room temperature and blocked with 5% BSA (bovine serum albumin) in TBST (Tris buffered saline containing Tween 20) for 5 min at room temperature. Slides were incubated with anti-mouse CD8a monoclonal antibody (clone 4SM15, eBiosciences, catalog #14-0808-82) at 5 µg/mL, followed by anti-rat IgG Biotin, Streptavidin HRP (anti-Rat Ig HRP Detection Kit, BD Biosciences, catalog #551013), and DAB visualization. Nuclei were counterstained with hematoxylin. Control slides were stained as above, except the primary anti-mouse CD8 antibody was omitted. Stained slides of interest were digitalized using a Path Finder Enabler IV Slide Scanner (Electron Microscopy Sciences).

### Statistical analyses

Data were analyzed using GraphPad Prism (Version 6.1, GraphPad Software, La Jolla, CA, USA). Student’s *t*-test was used when comparing two groups. When comparing multiple groups, one-way ANOVA tests, followed by Bonferroni corrections for multiple comparisons, were performed. All *p*-values correspond to two-tailed tests; significance was considered to be at *p* < 0.05.

### Ethics approval and consent to participate

Animal studies were approved by the Institutional Animal Care and Use Committee at the University of Alabama at Birmingham and performed in compliance with guidelines from the Public Health Service Policy and Animal Welfare Act of the United States.

## Results

### Radiolabeling efficiency of [^89^Zr]-malDFO-169 cDb

[^89^Zr]-malDFO-169 cDb was obtained in 82.5 ± 14.5% radiolabeling efficiency with 94.3 ± 0.2% radiochemical purity (n = 5 separate labeling reactions). The radiochemical purity remained above 94% after incubation for 1 day in phosphate-buffered saline (PBS) at 37 °C. This is consistent with previously reported studies^28^.

### In vitro characterization of [^89^Zr]-malDFO-169 cDb

The immunoreactivity of purified [^89^Zr]-malDFO-169 cDb with TK-1 cells was 85.3 ± 9.7% (n = 4 separate experiments). Less than 1.5% non-specific binding of [^89^Zr]-malDFO-169 cDb was observed with control cells that do not express CD8 (Supplementary Fig. 1). These results were similar to those observed in previous studies with radiolabeled conjugates of 169 cDb^28,31^.

### PET/CT imaging showed in vivo accumulation of [^89^Zr]-malDFO-169 cDb in orthotopic glioma tumors treated with oHSV

**Figure 2:**
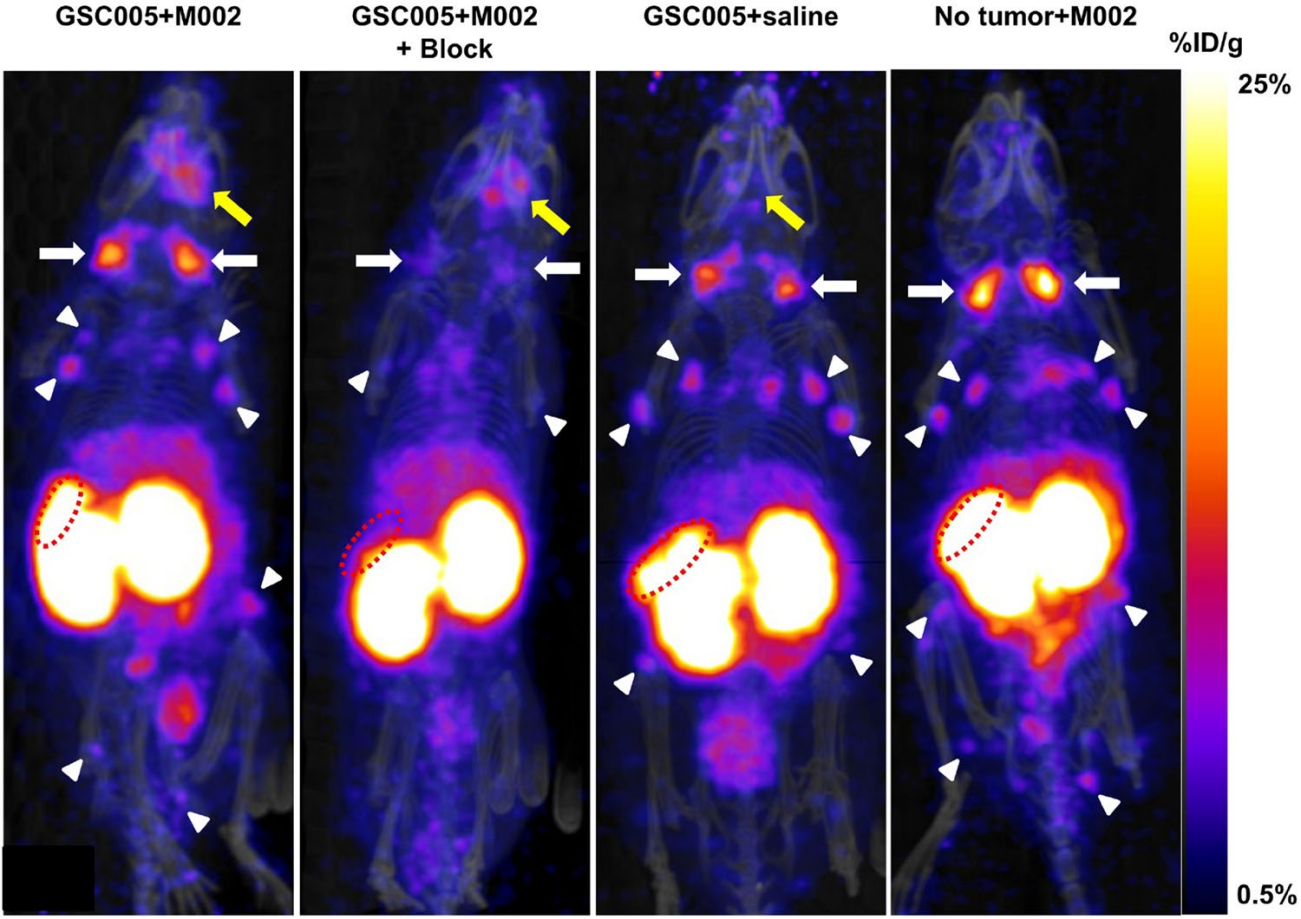
PET imaging of [^89^Zr]-malDFO-169 cDb shows accumulation in lymphoid tissues and clearance through the kidneys. Representative PET/CT maximum intensity projection (MIP) images acquired at 24 h after tail vein injection of [^89^Zr]-malDFO-169 cDb in mice from the GSC005 + M002 (left), GSC005 + M002 + block (middle-left), GSC005 + saline (middle-right), and no tumor + M002 (right) study groups. Retention of activity is apparent in the kidneys, spleens (dotted red ellipse), tumors (yellow arrows), cervical lymph nodes (white arrows), and systemic lymph nodes (white arrowheads). Notably reduced activity is apparent in the lymph nodes and spleens of mice in the GSC005 + M002 + block group relative to the other groups of mice. Images were generated using VivoQuant software (version 3.5patch 2, Invicro, LLC).

Representative PET/CT maximum intensity projection images acquired at 24 h post injection of [^89^Zr]-malDFO-169 cDb in mice from each study group treated with oHSV or saline control (GSC005 + M002, GSC005 + M002 + block, GSC005 + saline, no tumor + M002) are presented in Fig. 2. The tumor regions (SUV_mean_: 0.6; Table 1) and cervical lymph nodes (CLNs, SUV: 1.7) were clearly apparent at 24 h in the whole-body PET images of mice in the GSC005 + M002 group. The other systemic lymph nodes (axillary, inguinal, popliteal) showed relatively lower contrast (SUV < 1) relative to the CLNs during imaging. Activity from [^89^Zr]-malDFO-169 cDb also localized in the spleen (SUV > 6) of mice imaged at 24 h. At this time point, all normal, non-lymphoid tissues showed lower retention of activity (SUV < 2) relative to the kidneys (SUV > 8), which confirmed the primarily renal excretion route of the diabody as observed in prior reported studies^28^. Relative to the other study groups, mice in the GSC005 + M002 + block group showed notably reduced PET signal in the spleens and lymph nodes (Fig. 2).

**Table 1:** In vivo PET SUV results 24 h after injection of [^89^Zr]-malDFO-169 cDb.

Mouse group	SUV_mean_ ± SD		^a^SUV_max_ ± SD		^b^SUV_peak_ ± SD	
	Tumor	Contralateral hemisphere	Tumor	Contralateral hemisphere	Tumor	Contralateral hemisphere
GSC005 + M002	0.6 ± 0.16	0.1 ± 0.02	1.4 ± 0.43	0.4 ± 0.13	1.3 ± 0.39	0.3 ± 0.11
GSC005 + M002 + Block	0.6 ± 0.08	0.2 ± 0.03	1.4 ± 0.11	0.40 ± 0.06	1.3 ± 0.08	0.4 ± 0.07
GSC005 + saline	0.4 ± 0.13	0.2 ± 0.03	0.8 ± 0.31	0.35 ± 0.03	0.7 ± 0.28	0.3 ± 0.04
No Tumor + M002	0.4 ± 0.14^c^	0.4 ± 0.03	0.9 ± 0.24^c^	0.86 ± 0.11	0.8 ± 0.23^c^	0.8 ± 0.07

^a^Single voxel containing the maximum SUV value in the ROI.

^b^Average SUV of the 3 × 3 × 3 voxel grid containing the SUV_max_ voxel.

^c^Activity determined in the right brain hemisphere around the region of injection, rather than tumor region in the “No Tumor + M002” group of mice.

**Figure 3:**
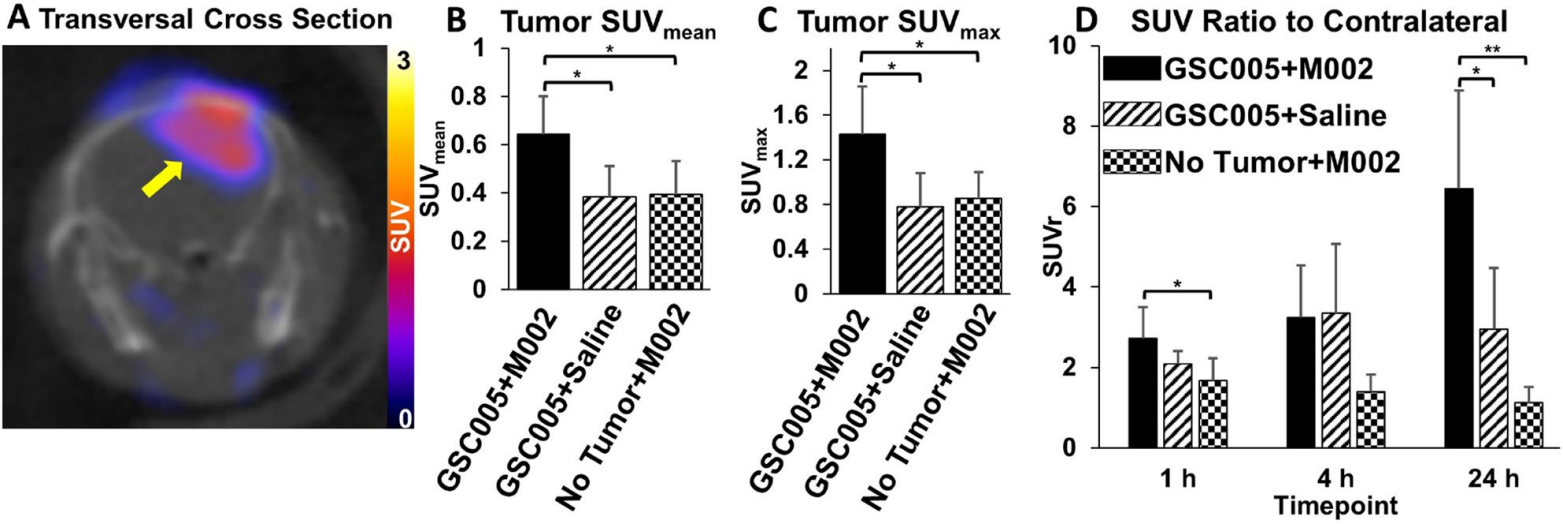
PET imaging shows increased accumulation of [^89^Zr]-malDFO-169 cDb in tumor bearing mice after M002 immunotherapy. (**a**) Representative PET/CT transversal cross section of a mouse in the GSC005 + M002 group showing retention of activity in the tumor (yellow arrow) at 24 h after tail vein injection of [^89^Zr]-malDFO-169 cDb. The image was generated using VivoQuant software (version 3.5patch 2, Invicro, LLC). (**b**) ROI analysis of the tumor region at 24 h shows significantly higher levels of activity (SUV_mean_) in mice with GSC005 tumors and M002 oHSV than with either alone. (**c**) Similarly, the SUV_max_ at 24 h was highest in the GSC005 + M002 group relative to the other groups. (**d**) The SUVratio (SUVr) between tumor-bearing brain and normal brain increased over time in the GSC005 + M002 group. Data in graphs are presented as group mean values and standard deviation. *p < 0.05, **p < 0.01 Graphs were generated using GraphPad Prism software (Version 6.1).

A representative PET/CT image indicating localization of activity in the tumor-bearing brain hemisphere in mice from the GSC005 + M002 group is shown in Fig. 3A. PET SUV data collected at 1, 4, and 24 h after injection of [^89^Zr]-malDFO-169 cDb in all groups of tumor-bearing mice indicated significantly higher accumulation of the radiotracer within the orthotopic tumors (average SUV_mean_ of all time points: 0.5 ± 0.2) relative to normal brain (average SUV_mean_ of all time points: 0.2 ± 0.1, p < 0.001) throughout the study period. The 24 h average PET SUV_mean_, SUV_max_, and SUV_peak_ values determined in the tumors and normal brain for each group of mice are provided in Table 1. As demonstrated in Fig. 3B,C, PET imaging at 24 h showed significantly higher tumor SUV_mean_ and SUV_max_ values in mice in the GSC005 + M002 group relative to the GSC005 + saline (SUV_mean_ p = 0.0207, SUV_max_ p = 0.0372) or no tumor + M002 (SUV_mean_ p = 0.0240, SUV_max_ p = 0.0343) control groups. Mice in the GSC005 + M002 group also showed the highest SUVr among all study groups. At the 24 h time point, the SUVr in the GSC005 + M002 group (6.4 ± 2.4) was significantly higher than the SUVr observed in the GSC005 + saline (3.0 ± 1.5, p < 0.05) or no tumor + M002 (1.1 ± 0.4, p < 0.01) groups (Fig. 3D). Representative 24 h PET/CT images of mouse brains from the GSC005 + M002 + block, GSC005 + saline, and no tumor + M002 groups are shown in Supplementary Fig. 2.

### Ex vivo biodistribution showed specific localization of [^89^Zr]-malDFO-169 cDb in lymphoid tissues

**Figure 4:**
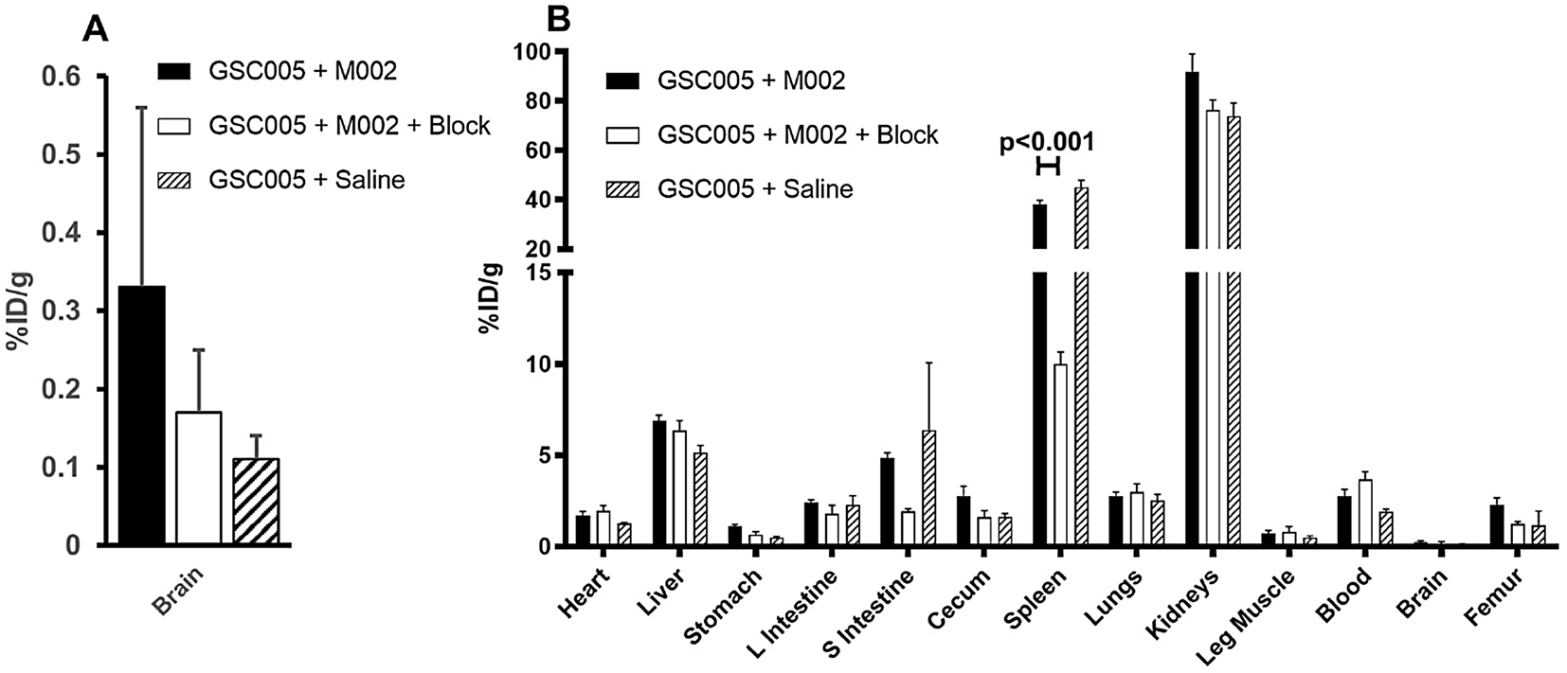
Biodistribution of [^89^Zr]-malDFO-169 cDb shows receptor-mediated accumulation in the spleen. (**a**) Ex vivo biodistribution data at 24 h shows uptake of activity in the whole brain was highest in the GSC005 + M002 group relative to the other groups. (**b**) Normal tissue biodistribution data at 24 h shows high uptake in the spleen and kidneys and low uptake in the brain. Splenic accumulation can be blocked by the co-administration of nonradioactive CD8 cys-diabody. Data in both graphs is presented as group mean values and standard deviation. Graphs were generated using Microsoft Excel version 15.0.

Following PET imaging, mice were euthanized for ex vivo analyses of tissue biodistribution of [^89^Zr]-malDFO-169 cDb (Fig. 4). While whole brain accumulation was low (Fig. 4A), the brains from mice in the GSC005 + M002 group showed the highest uptake of activity (0.33 ± 0.23%ID/g), with lower uptake in the brains from the other groups of mice (0.17 ± 0.08%ID/g and 0.11 ± 0.03%ID/g in the GSC005 + M002 + block and GSC005 + saline control groups, respectively), although these differences were not significant (ANOVA, p > 0.05). The brain-to-blood ratio, which is another metric to determine activity in the tumor-bearing tissue of interest, was significantly higher (p < 0.05) in the GSC005 + M002 group (0.15 ± 0.08) relative to the GSC005 + M002 + block group (0.05 ± 0.02) and the GSC005 + saline group (0.06 ± 0.01), but not relative to the no tumor + M002 group (0.09 ± 0.02; p > 0.05). Uptake of [^89^Zr]-malDFO-169 cDb in other tissues was consistent with the PET images (Fig. 2), indicating high accumulation in the spleen and kidneys and low retention in all other normal tissues; approximately 70% of the injected activity had cleared from all normal, non-lymphoid tissues and residual carcass at the time of biodistribution. Less than 4%ID/g [^89^Zr]-malDFO-169 cDb remained in the blood of all groups of mice at the time of biodistribution. Additionally, bone uptake at 24 h was low, indicating the stability of the ^89^Zr bond to the malDFO-169 cDb. The activity retained in spleens from mice in the GSC005 + M002 + block group (10%ID/g) was significantly lower (p < 0.001) relative to that observed in other groups (above 41%ID/g), indicating the specific retention of [^89^Zr]-malDFO-169 cDb in a CD8^+^ cell-rich tissue in these studies (Fig. 4B). Due to technical limitations, biodistribution analyses of lymph nodes were not achieved in these studies.

### Ex vivo analyses of brain tissues showed co-localization of [^89^Zr]-malDFO-169 cDb and CD8^+^ cells in orthotopic glioma tumors treated with oHSV

**Figure 5:**
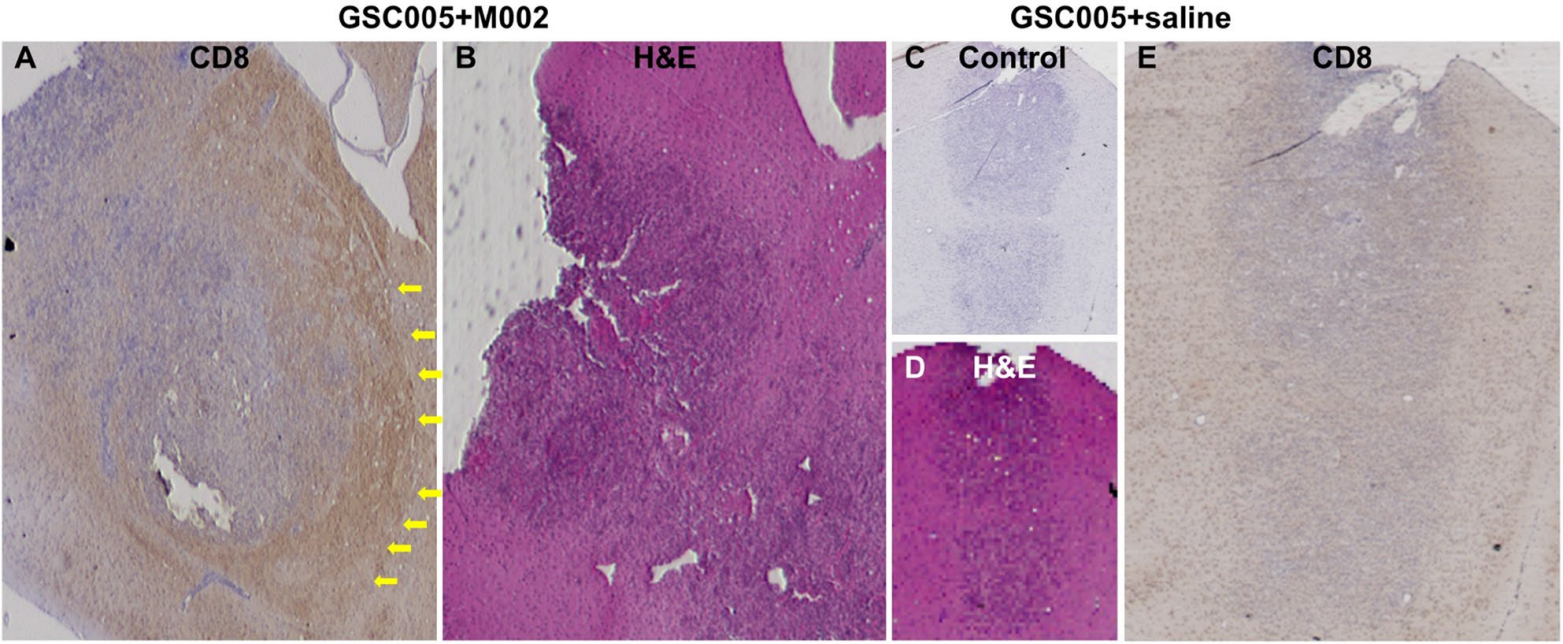
Ex vivo immunohistochemistry confirms CD8^+^ staining in tumor bearing mice after M002 immunotherapy. (**a**) Representative CD8 staining (4SM15) of tumor-bearing brain tissue sections from mice in the GSC005 + M002 group, with (**b**) corresponding H&E stain. Yellow arrows (**a**) indicate areas at the fringe of the tumor that stained positive for CD8. (**c**) Negative control stain without primary antibody, with (**d**) matching H&E, and (**e**) representative CD8 staining (4SM15) in tumor-bearing brain tissue sections from mice in the GSC005 + saline group. Non-adjacent tissue sections were used for H&E and CD8 staining.

Brain tissue autoradiography ROI analyses showed 4.3 ± 1.8 higher levels of activity from [^89^Zr]-malDFO-169 cDb in target brain regions that were confirmed to be tumor by H&E staining relative to levels of activity in contralateral normal brain (Supplementary Fig. 3). Immunohistochemistry showed more intense CD8^+^ staining in and around tumors from mice treated with oHSV (Fig. 5A,B) compared to mice treated with saline (Fig. 5C–E). These results support the link between oHSV immunotherapy, CD8^+^ cell recruitment, and specific retention of [^89^Zr]-malDFO-169 cDb in the orthotopic murine glioma tumors observed in these studies.

## Discussion

The studies presented provide preliminary evidence that CD8-targeted PET imaging can be used to evaluate immune response by detecting CD8^+^ cell infiltration into tumors during oHSV immunotherapy in orthotopic glioma tumors in syngeneic mice. The results demonstrated that an anti-mouse CD8 cDb labeled with ^89^Zr ([^89^Zr]-malDFO-169 cDb) specifically localized in orthotopic syngeneic glioma tumors in mice treated with the M002 oHSV compared to saline controls and contralateral brain tissue. These imaging studies indicate that there is an increase in CD8^+^ cell infiltration into the brain when treated with oHSV. Immunohistochemistry and autoradiography biologically validated these imaging findings and indicated the presence of CD8^+^ cells localized to the oHSV-treated tumors. Our data confirm that [^89^Zr]-malDFO-169 cDb is specifically retained in lymphatic tissues while the majority of activity clears from circulation and normal tissues within 24 h of systemic administration in mice treated with M002 oHSV. These imaging kinetics are consistent with prior studies that showed the circulation half-life of ^64^Cu-radiolabeled 169 cDb in mice was 62 min^31^, which is shorter than that of intact antibodies (> 2 days)^34,35^. Thus, sufficiently high tumor-to-background ratios for PET imaging with [^89^Zr]-malDFO-169 cDb were achieved within 24 h, which is earlier than what is usually optimal for intact antibody-based PET imaging (> 3 days after dosing)^36^. As the cDb used in these studies binds to murine rather than human CD8, the radiolabeled construct used in these preclinical studies is not amenable for clinical use. However, the findings from these studies have translational relevance, as oHSV immunotherapy is currently being evaluated in clinical trials in patients with glioma^19,37^, and CD8-targeted PET imaging studies using alternative targeting moieties are being performed in cancer patients treated with various types of immunotherapy^38,39^. The clinically approved oHSV talimogene laherparepvec in melanoma patients caused increased CD8^+^ T cell density in tumors which positively correlated with patient response to combinatorial oHSV and anti-PD-1 checkpoint immunotherapy^23^, indicating potential avenues to explore CD8-targeted PET imaging to assess responses to oHSV in models of melanoma and other solid tumors that undergo immunotherapy interventions. To our knowledge, this is the first study to utilize immune cell-targeted PET imaging approach to evaluate response to oncolytic virus therapy in glioma.

The patterns of activity retained in target brain regions of mice in the present studies are consistent with the hypothesis that M002 oHSV treatment mediated the specific retention of [^89^Zr]-malDFO-169 cDb in the treated glioma tumors. The very low uptake of [^89^Zr]-malDFO-169 cDb in normal brain regions was consistent with results from alternative radiolabeled polypeptide imaging agents that do not cross the BBB and show below 0.2% ID/g in normal brains of mice^40,41^. As all groups of mice received i.c. injections prior to i.v. administration of [^89^Zr]-malDFO-169 cDb, disruption of the blood–brain barrier (BBB) alone was not responsible for the higher PET signal observed in GSC005 tumor-bearing mice treated with M002 relative to the control groups (GSC005 + saline, no tumor + M002). BBB disruption from tumor growth and i.c. injection of the virus was anticipated to cause moderately elevated levels of [^89^Zr]-malDFO-169 cDb in the target brain hemisphere relative to the contralateral normal brain hemisphere, as was observed in GSC005 + M002 and control groups of mice. While the control group of mice given a blocking dose of the non-labeled diabody showed significantly reduced uptake of [^89^Zr]-malDFO-169 cDb in spleens relative to mice in the other groups based on ex vivo biodistribution analysis, the block dose failed to significantly lower activity in the tumors. This unexpected result could be due to an insufficient amount of non-labeled diabody to saturate all CD8 receptors, particularly in tissues with restricted access to macromolecules (e.g., lymph nodes, poorly vascularized tumors). Other CD8-targeted imaging studies used higher relative mass doses of the in vivo blocking agent (100 µg^42^) relative to the amount of unlabeled 169 cDb (65 µg) used in the present studies. However, the activity remaining in the blood of the blocked group in our study was higher than in the other groups (Fig. 4B) and resulted in a significantly lower brain-to-blood ratio relative to that observed in the GSC + M002 group. This result was expected due to slower CD8-mediated clearance of [^89^Zr]-malDFO-169 cDb from the blood in the blocked group of mice relative to the non-blocked groups, which were administered non-saturating doses of the radiolabeled diabody. These data support the hypothesis that CD8 targeting specifically mediated the update of [^89^Zr]-malDFO-169 cDb in the brains of tumor-bearing mice treated with M002.

We anticipated mice in the no tumor + M002 control group to exhibit some level of T cell response to oHSV, which would cause CD8^+^ cell-mediated localization of [^89^Zr]-malDFO-169 cDb in the M002-treated brain hemisphere even in the absence of tumor. As a Δ*γ134.5* oHSV, M002 replicates under control of the early-growth response-1 promoter^10^, which is expressed in proliferating tumor cells but is not expressed in the majority of normal, quiescent astrocytes. However, M002 is anticipated to transform low numbers of normal glia or microglia cells, causing these transformed cells to produce the T cell-attracting chemokine IL-12 (expressed from a constitutive promoter even in the absence of viral replication), which attracts CD8^+^ T cells. Our prior studies using M032, the human form of M002, in normal Aotus monkeys revealed increased inflammatory cell activity in M032-injected regions of the brain relative to the brains of monkeys administered vehicle or the alternative oHSV G207, which does not express IL-12^43^. Thus, small amounts of IL-12 from M002-treated mice were anticipated to cause intracranial localization of CD8^+^ T cells even when glioma cells are absent.

While FDG is the most widely used PET imaging agent, FDG shows limited specificity for distinguishing glioma progression from inflammatory or immune cell activity and has high uptake in normal brain. Therefore, radiolabeled amino acid analogs for PET imaging (e.g., l-[^11^C]methionine, *O*-(2-[^18^F]fluoro-ethyl)-l-tyrosine) are recommended for response assessment in glioma due to their higher specificity relative to FDG. Amino acid PET has shown potential to distinguish tumor response from treatment-related enhancement during *T*_1_ contrast or *T*_2_ MRI in glioma patients treated with immunotherapy^44^, although this approach has not yet been extensively evaluated in oHSV immunotherapy studies. Furthermore, these approaches provide information on metabolism of the tumor rather than immune cell infiltration. Targeted imaging of trans-gene expression in glioma during gene therapy delivered by viral vectors has been demonstrated in preclinical and clinical studies^45^. Our work adds to the growing body of studies that evaluate immunotherapy responses in vivo through immune cell-targeted PET imaging agents. Alternative CD8-targeted scaffolds (single-domain or F(ab)′2 antibody fragments) have shown correlations between PET signals and CD8^+^ cell infiltration as well as response to checkpoint immunotherapy in syngeneic murine non-CNS tumors^40,42,46,47^. A recent study showed that an anti-human CD8α-targeted minibody (IAB22M2C) labeled with ^64^Cu specifically localized in orthotopic patient derived xenograft GBM tumors implanted in brain striatum of humanized mice; the PET signal in the tumor directly correlated with the number of human CD8^+^ cells that had trafficked into the tumors after systemic injection of human peripheral blood monocytes into the humanized mice^41^. While that study did not investigate response to treatment, the findings support the rationale for non-invasive PET imaging to detect localization of CD8^+^ cells in intracranial glioma models. The cDb construct used in the present work is smaller than the minibody scaffold, which may result in differing pharmacokinetics and dosimetry. Furthermore cysteine residues in the cDb can be used for site-specific incorporation of the chelate for radiolabeling, as opposed to non-site-specific chelate linkage at lysine residues in the minibody construct, which may negatively impact immunoreactivity if linkage occurs within the epitope binding regions of the minibody. Granzyme B-targeted PET imaging is an alternative approach that enables detection of cytotoxic T cell activity rather than global population and correlates to melanoma and colon tumor responses during immunotherapy in preclinical studies^48–50^, although this imaging approach has not yet been used in glioma models. Another PET imaging strategy using radiolabeled substrates for deoxycytidine kinase, which is differentially expressed in proliferating T cells, has demonstrated potential for evaluating response during dendritic cell immunotherapy of glioma in preclinical and clinical studies^51^. Prior studies using radiolabeled 169 cDb in syngeneic murine colon and mammary tumors treated with anti-PD-1, anti-CD137, or CpG oligonucleotide immunotherapy showed roughly twofold higher uptake of the diabody in treated tumors relative to non-treated control tumors. The activity of radiolabeled 169 cDb in these tumors localized to regions with CD8^+^ cell infiltration and correlated with response, except in tumors with large necrotic regions due to treatment efficacy^28,31^. Our in vivo PET SUV results similarly indicated approximately twofold higher retention of the radiolabeled 169 cDb in intracranial glioma tumors treated with oHSV relative to non-treated or non-tumor control groups of mice, although our studies did not examine quantitative correlations between tumor infiltrating CD8^+^ cells and [^89^Zr]-malDFO-169 cDb retention as a function of oHSV treatment. Our PET images and autoradiography results, combined with the known pattern of CD8^+^ T cell localization in oHSV-treated glioma tumors, suggest that the tumor uptake of [^89^Zr]-malDFO-169 cDb observed in M002-treated mice was mediated by CD8^+^ cells in response to oHSV immunotherapy.

Various types of oHSV and combinatorial immune therapy strategies have been shown to effectively eradicate glioma tumors in significant percentages of mice in preclinical studies, although some mice in these treatment groups were resistant to treatment and displayed rapid tumor progression^12,52–54^. Characterizing the in vivo immune response or identifying additional biomarkers at the start of treatment that correlate to long term survival following oHSV treatment would provide novel prognostic information to guide therapy strategies^47^. Furthermore, better understanding of the kinetics of CD8^+^ cell infiltration in the brain could provide biological information to help guide multi-dose and combination therapies. Currently, those data are limited to biodistribution studies requiring different cohorts of mice for each time point of interest. Targeted imaging of the in vivo immune cell response to oHSV in glioma has not been widely explored. A recent report showed moderate yet significant correlations between MRI tumor volume measurements 6–7 days after intratumoral oHSV treatment and percentages of tumor antigen-specific activated CD8^+^ T cells measured ex vivo by flow cytometry the day after MRI assessment^13^. While several preclinical studies in glioma models treated with oHSV M002 showed higher numbers of CD8^+^ cells relative to non-treated control tumors^11,55^, studies using alternative oHSV constructs showed no significant differences in numbers of CD8^+^ tumor infiltrating lymphocytes between treatment and control groups^13^. This could be due to differences in intrinsic immunogenicity in tumor models and to differing actions of the oHSV constructs evaluated (e.g., differences in cytokine release). Such factors are anticipated to influence the signals observed during response assessment with CD8-targeted imaging agents. While preclinical studies can provide insights into mechanisms of immune responses during treatment, preclinical models are not able to predict clinical response to immunotherapy or other treatments in human patients with glioma.

It was beyond the scope of the pilot studies presented above to evaluate if [^89^Zr]-malDFO-169 cDb PET imaging can predict long-term tumor response to M002 oHSV treatment in orthotopic glioma models. However, previous animal studies indicate that M002 oHSV is effective in these tumor models^10,11,18,55^. There are several limitations to our studies. We did not include in vivo assessment of tumor size. Future work could utilize longitudinal PET and MRI to assess correlations between PET images and glioma tumor response to oHSV immunotherapy in individual animals. Perfusion of brains prior to resection and radiography analyses could allow for further biological analysis in the future by reducing non-specific blood pooling in the tumors in ex vivo biodistribution and autoradiography analyses. Further, future work could employ a CD8-depleting antibody in oHSV treated cohorts to further verify the CD8^+^ cell-mediated localization of the PET signal in glioma tumors treated with oHSV. Additionally, due to restrictions associated with residual radioactivity, we did not have the capability to analyze the individual immune cell populations by flow cytometry in the animals that were injected with [^89^Zr]-malDFO-169 cDb. Future studies could use a parallel group of tumor-bearing mice treated with M002 to quantify the infiltrating CD8^+^ cell population by flow cytometry and potentially explore kinetics of CD8^+^ cell infiltration, albeit this requires many mice and does not allow for longitudinal evaluation in the same mouse. The CD8-targeted diabody used in our studies is anticipated to bind to the global CD8^+^ cell population regardless of the cells’ antitumor activity or exhaustion status. Studies have indicated that macrophages and other inflammatory immune cell populations besides CD8^+^ T cells contribute to the antitumor response to oHSV in preclinical models^14,52^. Therefore, it would be of interest to evaluate alternative imaging agents that target these cell populations during future oHSV immunotherapy studies in glioma models. The inability of the CD8-targeted diabody to cross the BBB is a potential limitation for long-term translation of this PET imaging approach in glioma tumor regions where the BBB remains intact. Additional studies are needed to determine if PET signals from the diabody in glioma tumors with BBB disruption correlates to response to oHSV immunotherapy.

## Conclusions

The results from these studies support the concept that [^89^Zr]-malDFO-169 cDb can be used for CD8-targeted in vivo PET imaging in immune-competent mice bearing orthotopic glioma tumors treated with oHSV. Specific retention of [^89^Zr]-malDFO-169 cDb in oHSV-treated tumors relative to controls and normal brain was observed over a 24 h imaging period. Ex vivo autoradiography and tissue immunohistochemistry analyses demonstrated concordance between tumor location, radioactivity and CD8^+^ cell localization following oHSV treatment. These preliminary results provide first of its kind proof-of-concept data which support future work using CD8-targeted PET imaging to assess responses to oHSV and potentially additional types of immunotherapy in glioma models.

## Supplementary Information

Supplementary Information.

## Abbreviations

ANOVA: Analysis of variance
BBB: Blood–brain barrier
cDb: Cys-diabody
CLN: Cervical lymph node
CNS: Central nervous system
CT: Computed tomography
FDG: 2-Deoxy-2[^18^F]fluoro-d-glucose
FBS: Fetal bovine serum
H&E: Hematoxylin and eosin
%ID/g: Percent injected dose per gram
malDFO: Maleimide-desferrioxamine
MRI: Magnetic resonance imaging
oHSV: Oncolytic herpes simplex virus
PET: Positron emission tomography
PD-1: Programmed death-1
PFU: Plaque-forming units
ROI: Region of interest
SUV: Standardized uptake value
SUVr: SUV ratio
TBST: Tris buffered saline Tween 20
