# Author Correction: Positron emission tomography imaging with ^89^Zr-labeled anti-CD8 cys-diabody reveals CD8^+^ cell infiltration during oncolytic virus therapy in a glioma murine model

**DOI:** 10.1038/s41598-021-00042-x

**Published:** 2021-10-08

**Authors:** Benjamin B. Kasten, Hailey A. Houson, Jennifer M. Coleman, Jianmei W. Leavenworth, James M. Markert, Anna M. Wu, Felix Salazar, Richard Tavaré, Adriana V. F. Massicano, G. Yancey Gillespie, Suzanne E. Lapi, Jason M. Warram, Anna G. Sorace

**Affiliations:** 1 Department of Neurosurgery, University of Alabama at Birmingham, Birmingham, AL USA; 2 Department of Radiology, University of Alabama at Birmingham, Volker Hall G082, 1670 University Boulevard, Birmingham, AL 35294 USA; 3 O’Neal Comprehensive Cancer Center, University of Alabama at Birmingham, Birmingham, AL USA; 4 Department of Immunology and Theranostics, City of Hope, Duarte, CA USA; 5 Department of Molecular and Medical Pharmacology, Crump Institute for Molecular Imaging, David Geffen School of Medicine at University of California Los Angeles, Los Angeles, CA USA; 6 Regeneron Pharmaceuticals, Inc., Tarrytown, NY USA; 7 Department of Otolaryngology, University of Alabama at Birmingham, Volker Hall G082, 1670 University Boulevard, Birmingham, AL 35294 USA; 8 Department of Biomedical Engineering, University of Alabama at Birmingham, Birmingham, AL USA

Correction to: *Scientific Reports* 10.1038/s41598-021-94887-x, published online 28 July 2021

The Acknowledgements section in the original version of this Article was incomplete.

“Yolanda Hartman, Sheila Bright, Catherine Langford, Sharon Samuel, Erika McMillian, Brian Wright, Lauren Radford, Charlotte Jeffers, Jennifer Bartels, Tolulope Aweda, Jonathan McConathy, Jinda Fan, Norio Yasui, Dattatray Devalankar, Himani Modi, Morgan Richardson, and staff of the University of Alabama Cyclotron Facility are gratefully acknowledged for their contributions and informative discussions. Funding was provided by the National Institutes of Health/National Institute of Neurological Disorders and Stroke T32 UAB Training Program in Brain Tumor Biology (T32 NS048039), the UAB Brain Tumor Core Facility (USPHS NCI P20CA151129), the National Center for Advancing Translational Research of the National Institutes of Health (UL1TR001417), the American Cancer Society (RSG-18-006-01-CCE), National Cancer Institute (R01CA24058) and the Comprehensive Cancer Center at UAB (NIH P30CA013148). JWL is supported by funding from the Department of Defense (W81XWH-18-1-0315) and National Institutes of Health (R01AI148711). JMW is supported by the Department of Defense (RCRP CA170769). The content is solely the responsibility of the authors and does not necessarily represent the official views of the National Institutes of Health.”

now reads:

“Yolanda Hartman, Sheila Bright, Catherine Langford, Sharon Samuel, Erika McMillian, Brian Wright, Lauren Radford, Charlotte Jeffers, Jennifer Bartels, Tolulope Aweda, Jonathan McConathy, Jinda Fan, Norio Yasui, Dattatray Devalankar, Himani Modi, Morgan Richardson, and staff of the University of Alabama Cyclotron Facility are gratefully acknowledged for their contributions and informative discussions. Funding was provided by the National Institutes of Health/National Institute of Neurological Disorders and Stroke T32 UAB Training Program in Brain Tumor Biology (T32 NS048039), the UAB Brain Tumor Core Facility (USPHS NCI P20CA151129), the National Center for Advancing Translational Research of the National Institutes of Health (UL1TR001417), the American Cancer Society (RSG-18-006-01-CCE), National Cancer Institute (R01CA24058). This was supported by the Preclinical Imaging Facility at the O’Neal Comprehensive Cancer Center at UAB (NIH P30CA013148). JWL is supported by funding from the Department of Defense (W81XWH-18-1-0315) and National Institutes of Health (R01AI148711). The content is solely the responsibility of the authors and does not necessarily represent the official views of the National Institutes of Health.”

The original Article has been corrected.

