# Supplementary material for: Positron emission tomography imaging with 89Zr-labeled anti-CD8 cys-diabody reveals CD8+ cell infiltration during oncolytic virus therapy in a glioma murine model
Source: Sci Rep. 2021 Jul 28;11:15384. doi: 10.1038/s41598-021-94887-x (PMC8319402; doi:10.1038/s41598-021-94887-x)

**Supplementary Information**

**^89^Zr-labeled anti-CD8 cys-diabody PET reveals CD8^+^ cell infiltration during oncolytic virus therapy in a glioma murine model: Preliminary study**

Benjamin B. Kasten^1+^, Hailey A. Houson^2+^, Jennifer M. Coleman^1^, Jianmei W. Leavenworth^1,3^, James M. Markert^1,3^, Anna M. Wu^4,5^, Felix Salazar^5^, Richard Tavaré^6^, Adriana V. F. Massicano^2^, G. Yancey Gillespie^1,3^, Suzanne E. Lapi^2,3^, Jason M. Warram^7,3^*, Anna G. Sorace^2,3,8^*

^1^Department of Neurosurgery, University of Alabama at Birmingham, Birmingham, AL, United States.

^2^Department of Radiology, University of Alabama at Birmingham, Birmingham, AL, United States.

^3^O’Neal Comprehensive Cancer Center, University of Alabama at Birmingham, Birmingham, AL, United States.

^4^Department of Immunology and Theranostics, City of Hope, Duarte, CA, United States

^5^Crump Institute for Molecular Imaging, Department of Molecular and Medical Pharmacology, David Geffen School of Medicine at University of California Los Angeles, Los Angeles, CA, United States.

^6^Regeneron Pharmaceuticals, Inc., Tarrytown, NY, United States.

^7^Department of Otolaryngology, University of Alabama at Birmingham, Birmingham, AL, United States.

^8^Department of Biomedical Engineering, University of Alabama at Birmingham, Birmingham, AL, United States.

^+^These authors contributed equally to this work.

***Corresponding Authors**:

Jason M. Warram, PhD

Department of Otolaryngology

University of Alabama at Birmingham,

Volker Hall G082

1670 University Boulevard

Birmingham, AL 35294, USA

Telephone: 1-205-996-5009

Fax: 1-205-975-6522

Email: [mojack@uab.edu](mailto:mojack@uab.edu)

Anna G. Sorace, PhD

Department of Radiology

University of Alabama at Birmingham,

Volker Hall G082

1670 University Boulevard

Birmingham, AL 35294, USA

Telephone: 1-205-934-3116

Fax: 1-205-975-6522

Email: [asorace@uabmc.edu](mailto:asorace@uabmc.edu)

**Running title**: CD8 PET imaging of virus immunotherapy in glioma

**Supplementary Figure 1:** *In vitro* immunoreactivity of [^89^Zr]-malDFO-169 cDb showing specific binding to CD8^+^ TK-1 cells and very low binding to CD8^-^ control cells. Data is shown as mean values and standard deviations of four independent experiments.


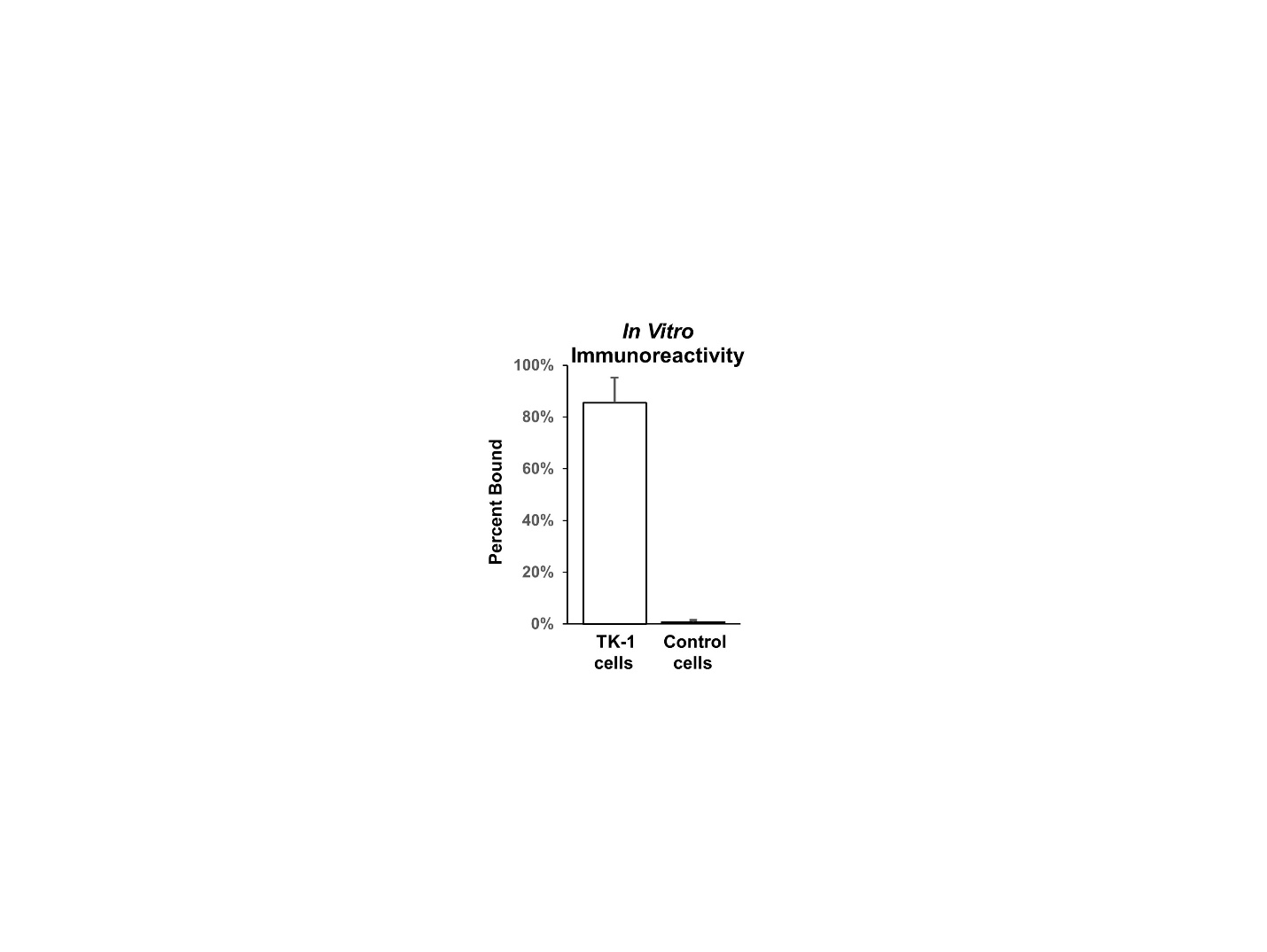


**Supplementary Figure 2:** Representative PET/CT transversal cross section images of brains from mice in the GSC005+M002+block, GSC005+saline, and no tumor+M002 groups at 24 h after tail vein injection of [^89^Zr]-malDFO-169 cDb. Retention of activity in the tumor (yellow arrow) is low in these control groups of mice. White arrowheads indicate areas of extra-cranial wound tissue resulting from the i.c. procedures.


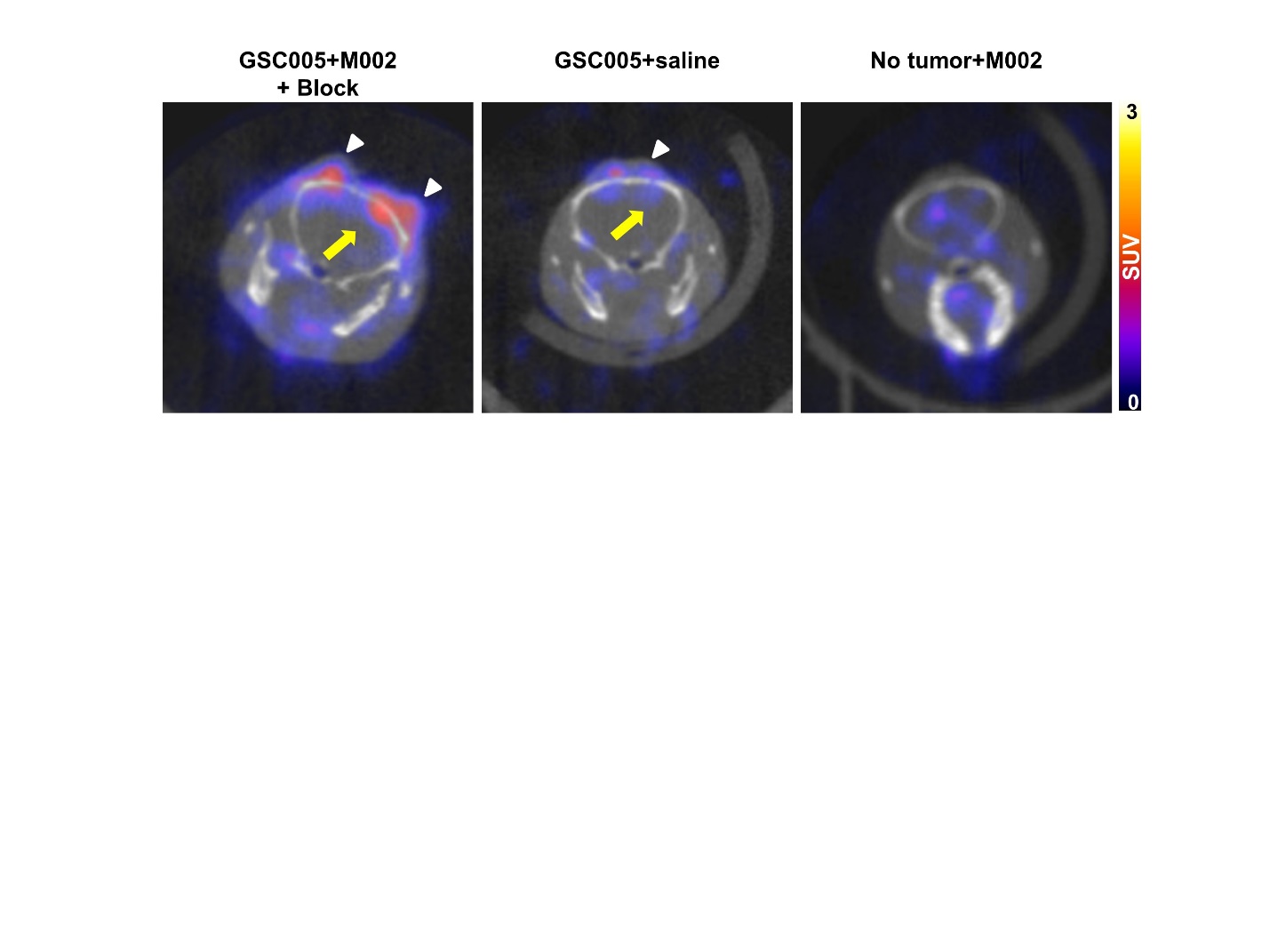


**Supplementary Figure 3:** (**a**) *Ex vivo* autoradiography and (**b**) corresponding H&E staining of serial 1 mm brain tissue sections from a mouse in the GSC005+M002 group. Blue color in (**a**) corresponds to lower radioactivity while red color corresponds to higher radioactivity.


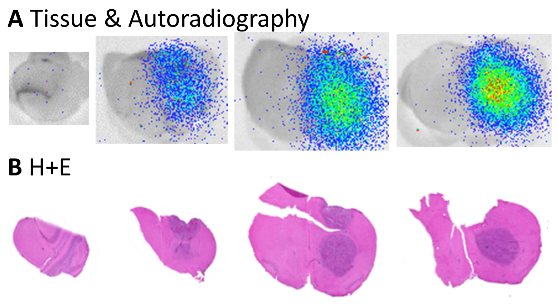

Supplement: Supplementary file 1 — Supplementary Information. [file 41598_2021_94887_MOESM1_ESM.docx]
